# Corrigendum for: “Oomycete-specific ITS primers for identification and metabarcoding” published in MycoKeys, doi: 10.3897/mycokeys.14.9244

**DOI:** 10.3897/mycokeys.41.30558

**Published:** 2018-11-05

**Authors:** Taavi Riit, Leho Tedersoo, Rein Drenkhan, Eve Runno-Paurson, Harri Kokko, Sten Anslan

**Affiliations:** 1 Institute of Ecology and Earth Sciences, University of Tartu, Ravila 14a, 50411 Tartu, Estonia; 2 Institute of Forestry and Rural Engineering, Estonian University of Life Sciences, Kreutzwaldi 5, 51014 Tartu, Estonia; 3 Institute of Agricultural and Environmental Sciences, Estonian University of Life Sciences, Kreutzwaldi 5, 51014 Tartu, Estonia; 4 Department of Environmental and Biological Sciences, University of Eastern Finland, P.O. Box 1627, FI-70211 Kuopio, Finland

The oomycete-specific ITS primers published by Riit et al. (2016) have been put to use in the scientific community working with oomycetes. Recently, however, it has been brought to our attention that the sequences of the primers ITS1oo and ITS3oo shown in the first Figure of the published manuscript are incomplete, when compared to the sequences of the same primers as listed on the UNITE website. This discrepancy is derived from re-checking primer sequences from tube labels that are restricted to the first 18 bases.

Closer examination revealed that the sequence of primer ITS1oo in Figure 1 is missing one nucleotide from the 3’ end and the primer ITS3oo is missing two nucleotides from the 3’ end. These errors are expected to reduce relative primer specificity to Oomycetes, which probably results in a lower proportion of this group in metabarcoding studies. We hereby provide the updated figure (Figure 1) with correct information. We apologise to all users of these erroneous primers for their suboptimal performance. We are grateful to Dr. Diana Marčiulynienė and Dr. Sannakajsa Velmala for identifying these problems.

**Figure 1.**
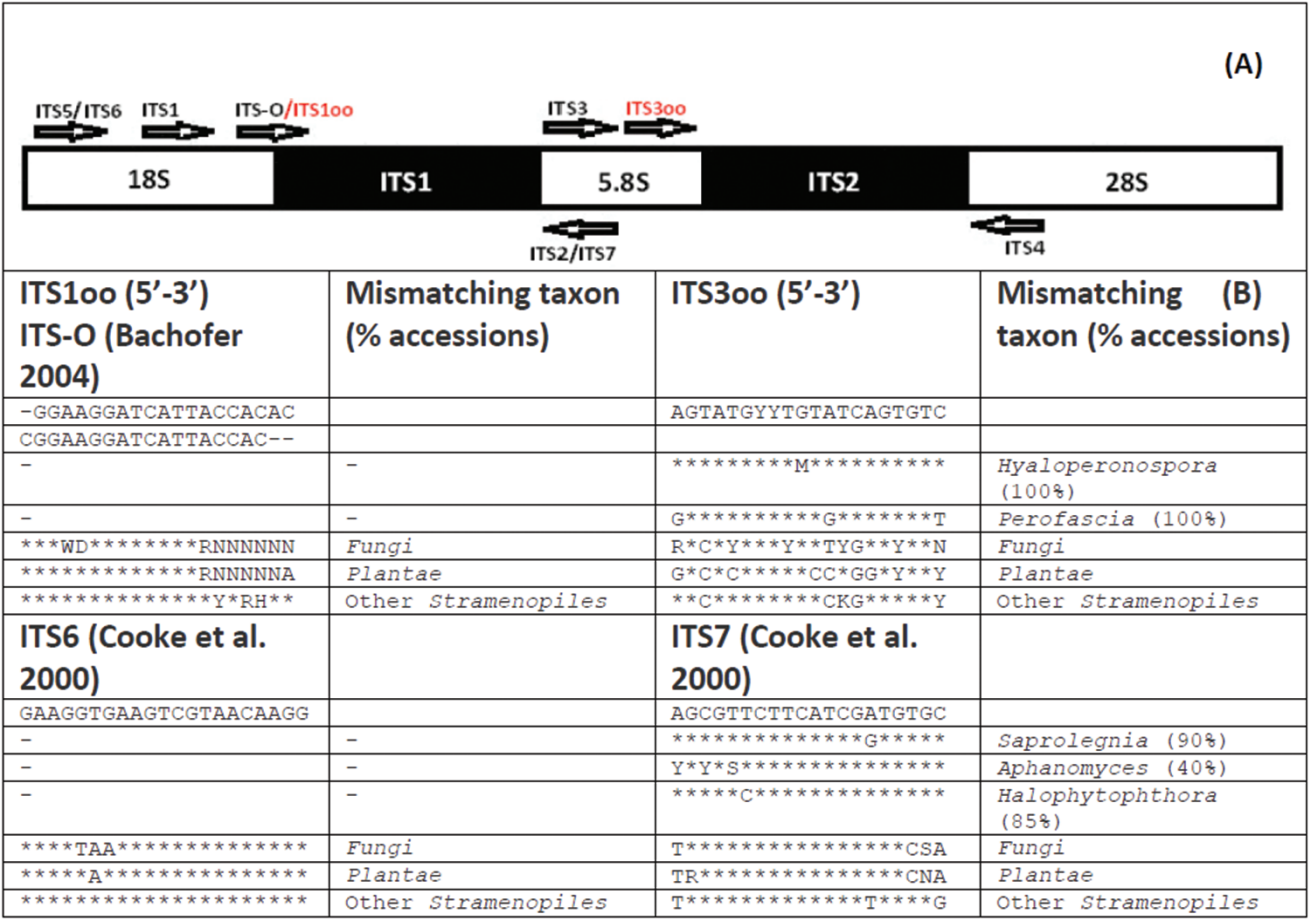
**A** Map of universal and oomycete-specific ITS region primers **B** Taxa with mismatches in the binding sites of primers ITS1oo and ITS3oo. Only taxa with 10% or more mismatching accessions are shown.
